# STAT3 Signaling Induces the Differentiation of Human ICOS^+^ CD4 T Cells Helping B lymphocytes

**DOI:** 10.1371/journal.pone.0071029

**Published:** 2013-07-26

**Authors:** Laure Ysebrant de Lendonck, Fouad Eddahri, Yves Delmarcelle, Muriel Nguyen, Oberdan Leo, Stanislas Goriely, Arnaud Marchant

**Affiliations:** 1 Institute for Medical Immunology (IMI), Université Libre de Bruxelles, Charleroi, Belgium; 2 ImmuneHealth, Charleroi, Belgium; Maisonneuve-Rosemont Hospital, Canada

## Abstract

The generation of high-affinity antibodies and the development of B cell memory are dependent on the help provided by CD4 T cells. Mouse studies indicate that STAT3 signaling in CD4 T cells promotes the acquisition of the B cell help function. However, the role of STAT3 in humans has been controversial. In this study, we show that IL-6 and other STAT3 activating cytokines (IL-21 and IL-27) induce the differentiation of CD4 T cells promoting antibody production by B cells. The acquisition of B cell stimulating properties by naive cord blood CD4 T cells required the STAT3-dependent expression of ICOS and IL-21. Gene reporter and ChIP experiments unambiguously demonstrated that upon IL-6 stimulation, STAT3 induces the transcription of the *ICOS* gene through direct recruitment to the proximal promoter region indicating that STAT3 acts in part through the direct activation of the ICOS gene.

## Introduction

The generation of high affinity antibodies and the development of B cell memory are largely dependent on the help provided by CD4 T cells [1]. The B cell help function was long thought to be attributable to the Th2 subset. This notion was based on the ability of Th2 derived cytokines, in particular IL-4, to sustain B cell growth, differentiation and isotype switch [2], [3]. More recently, follicular helper CD4 T (T_FH_) cells, originally described in germinal centers (GCs) within human tonsils, have been established as a critical subset promoting B cell responses [4], [5], [6].

Functional differentiation of CD4 T cells is dependent on the cytokine driven activation of specific members of the signal transducer and activator of transcription (STAT) family [7], [8], [9], [10], [11]. Studies in mice indicate that STAT3 signaling induces the acquisition of B cell help properties by CD4 T cells, both *in vitro* and *in vivo* [12], [13], [14], [15]. STAT3 is the major signaling molecule for IL-6 and IL-21 and double inactivation of IL-6 and IL-21 is associated with decreased frequencies of T_FH_ cells in mice [14]. In humans, two studies indicated that IL-12 promotes the acquisition of B cell help capacity by CD4 T cells through the activation of STAT4 and that STAT3 signaling may be less critical in this process [16], [17]. However, naive CD4 T cells from patients with STAT3 mutations causing autosomal dominant hyper-IgE syndrome (AD-HIES) were recently shown to be unable to acquire B cell help activity when stimulated in the presence of STAT3 activating cytokines [18].

Multiple mechanisms account for the capacity of CD4 T cells to provide help to B cells [19]. IL-21 was identified as the most potent cytokine driving plasma cell differentiation [20], [21], [22], [23]. Several surface molecules, including inducible costimulator (ICOS), CD40L, and SLAM associated protein (SAP) are involved in the interaction between T and B lymphocytes [5], [24], [25]. ICOS seems to play a central role in the B cell help function of CD4 T cells by providing essential signals for the initiation and maintenance of antibodies production. ICOS deficiency is associated with the loss or a defective formation of germinal centers in mice and humans [26], [27]. Recently, ICOS was shown to be required for the early differentiation of T_FH_ cells during mouse LCMV infection [28]. Finally, both IL-21 and ICOS were shown to be involved in the B cell help activity of CD4 T cells differentiated in the presence of IL-12 [16].

In the present study, we unequivocally show that STAT3 activating cytokines (IL-6, IL-21 and IL-27) can promote the acquisition of B cell help function by naive cord blood CD4 T cells in an IL-21/ICOS dependent way. Furthermore, we show that ICOS expression is directly regulated at the transcriptional level by STAT3 in primary CD4 T lymphocytes.

## Materials and Methods

### Ethics statement

The study was approved by the ethics committee of the Faculty of Medicine of the Université Libre de Bruxelles. Adult blood was obtained from healthy volunteers following written informed consent and umbilical cord blood samples were collected following written parental consent from healthy full term neonates at the department of Obstetrics and Gynecology of four hospitals (Clinique Notre Dame-Charleroi, Clinique Notre Dame de Grâce-Gosselies, CHU-Charleroi and CHU-Tivoli-La Louvière).

### Isolation of CD4 T cells and B cells

Naive CD4 T cells were isolated from cord blood mononuclear cells using Naive CD4^+^ T Cell Isolation Kit II (Miltenyi). B cells were purified from adult PBMCs by positive selection using CD19 MicroBeads (Miltenyi). T and B cell purity was consistently above 95%, as indicated by flow cytometry analysis of CD4 and CD45RA or CD19 expression, respectively.

### Dendritic cell generation and stimulation

Monocytes were isolated from adult PBMCs by positive selection using CD14 microbeads (Miltenyi). Dendritic cells (DCs) were generated by culturing monocytes with IL-4 (500 U/ml) and GM-CSF (800 U/ml) (Gentaur). At day 6, DCs were stimulated with LPS (10 µg/ml, Invivogen) or Poly(I∶C) (10 µg/ml, GE Healthcare). Culture supernatants were harvested after 18 h of stimulation.

### Stimulation of naive CD4 T cells via CD3 and CD28

Naive cord blood CD4 T cells (2×10^6^ cells/well) were stimulated during 72 hours with plate bound anti-CD3 mAb (5 μg/ml, OKT3) and soluble anti-CD28 mAb (1 μg/ml, CD28.2 clone, BD biosciences) in flat bottom 24 well plates in AIMV® medium conditioned with recombinant cytokines (rIL-4, rIL-6 and rIL-12 at 25 ng/ml; rIL-27, IL-21 at 50 ng/ml) (R&D systems). In some experiments, supernatants from activated DCs were used instead of recombinant cytokines and were conditioned with neutralizing anti-IL-6 (10 μg/ml, eBioscience) or anti-IL-12p70 (10 μg/ml, R&D systems) mAbs.

### Analysis of cytokine production, cell phenotype and STAT3 activation

For intracellular cytokine staining, naive cord blood CD4 T cells stimulated for 3 days were restimulated with PMA (25 ng/ml) and ionomycin (1 μg/ml) for 5 hours in the presence of GolgiStop (BD biosciences) for the last 4 hours. Cells were then fixed, permeabilized and the cytokines expressed in the cytoplasm were analyzed with anti-IL-21 PE (eBioscience) and anti-IFN-γ PB mAbs (BD biosciences). Cell phenotype was characterized by surface staining with the following antibodies: ICOS FITC (eBioscience), CD69 PE-Cy5 (BD Biosciences), CXCR5 AF 488 (BD biosciences). For detection of STAT phosphorylation, cells were fixed with Cytofix buffer and incubated in Perm III solution following manufacturer's protocol (BD biosciences). Cells were stained with p(Y705) STAT3 APC mAb (BD biosciences). For Bcl-6 detection, cells were fixed and permeabilized with the Foxp3/Transcription factor staining buffer set (eBioscience) and stained with Bcl-6 APC mAb (eBioscience). Data were obtained on a Cyan ADP LX9 cytometer (DakoCytomation) and analyzed using the FlowJo 9.4 software (TreeStar).

### Determination of Cytokine levels from activated CD4 T cells

For analysis of cytokine secretion, naive cord blood CD4 T cells stimulated for 3 days were restimulated for 3 days with plate bound CD3 mAb in flat bottom 96 well plates (1×10^5^ cells/well) in RPMI complete medium with 10% FBS to measure IFN-γ, IL-10 and IL-21 production by ELISA (Ready-SET-Go!® from eBioscience).

### RNA purification and real time RT-PCR

Total RNA was extracted using a MagnaPure LC RNA-High Performance Isolation Kit (Roche Diagnostics, Brussels, Belgium). RT and real time PCR reactions were then carried out using LightCycler-RNA Master Hybridization Probes (one step procedure) on a Lightcycler 480 apparatus (Roche Diagnostics). Primer and probe sequences are available upon request.

### Co-culture of T and B cells

Activated cord blood CD4 T cells (1×10^5^ cells/well each) were irradiated (2000 rads) and co-cultured in 96 well flat bottom plates with heterologous adult B cells (1×10^5^ cells/well each) in RPMI complete medium containing 10% FBS and either plate bound anti-CD3 (5 µg/ml, OKT3) or TSST (200 ng/ml, Sigma Aldrich). In some experiments, ICOS-L/mIgFc, IL-21R/Fc or IgG1Fc (R&D systems) were added to the culture. Igs (IgM and IgG) concentrations were measured in culture supernatants at day 7 by ELISA.

### STAT3 knockdown

Purified naive cord blood CD4 T Cells were incubated in Accell siRNA delivery media with rIL-2 (10 I.U/ml, R&D systems) and 1 µM STAT3 or non targeting control siRNA Accell SMART pool from Dharmacon (ABgene, Epsom, UK). 48 hours after transfection, knockdown efficiency was assessed by qRT-PCR and cells were used in further experiments. T cell viability was assessed before co-cultures with B cells by flow cytometry (violet LIVE/DEAD viability/cytotoxicity assay kit, Invitrogen) or by trypan blue staining.

### Plasmid constructs

A 705 bp fragment of the ICOS gene (nucleotide [nt] –684/+20) was amplified by PCR from human genomic DNA and subsequently cloned into the pCR2.1 vector by TA cloning (Invitrogen). The insert was subcloned into the pGL3-BASIC vector (Promega) as a HindIII-XhoI insert to generate the luciferase reporter plasmid. Deletion mutants of the 5′ flanking regions were generated by PCR and cloned into pGL3-BASIC as Kpn I-Bgl II fragments. The −174/+20 construct was used as a template for mutagenesis by the QuickChange Site-directed Mutagenesis Method (Agilent technologies). All constructs were fully resequenced prior to use. pBabe-human STAT3C expression vector was kindly provided by C. Horvath (Northwestern University, Evanston, Illinois).

### Transient transfection and luciferase assays

EL4 mouse thymoma T cells were transfected using FuGENE-6 (Roche Diagnostics). Promoter activities were analyzed 48 hours after transfection using the Dual-Glo Luciferase Reporter Assay system (Promega). Promoter activities were then normalized to Renilla luciferase activities.

### Chromatin Immunoprecipitation (ChIP)

ChIP experiments were performed on naive cord blood CD4 T cells. 2×10^7^ cells were stimulated for 60 min with plate bound anti-CD3 in the presence or absence of recombinant cytokine as indicated. Cells were cross-linked by 1% formaldehyde for 10 min at RT and reaction was stopped by addition of glycine. Cells were resuspended in ChIP lysis buffer and subjected to sonication (40 cycles of 30 sec) using a bioruptor device (Diagenode, Liège, Belgium) to obtain DNA fragments ranging from 150 to 500 bp. Chromatin fractions were precleared with protein G-magnetic beads (ActiveMotif) followed by immunoprecipitation overnight at 4°C with 2 µg of anti-STAT3 (clone C20, Santa Cruz) or its control antibody. After 3 washes and elution, cross-linking was reversed for 4 hours at 65°C and DNA was purified and subjected to qPCR. Primer and probe sequences are available upon request.

### Statistics

Data are presented as individual results or means and standard errors on the mean. Multiple parameter comparisons were performed with the one-way ANOVA test. When significant differences were observed, data were compared using the paired Wilcoxon signed-rank test or the paired Student's t-test when the sample size was lower than 6. Statistical significance was defined at p values below 0.05. GraphPad Prism 5 was used to perform the analyses.

## Results

### IL-6, IL-12 and IL-27 promote the acquisition of B cell help capability

**Figure 1:**
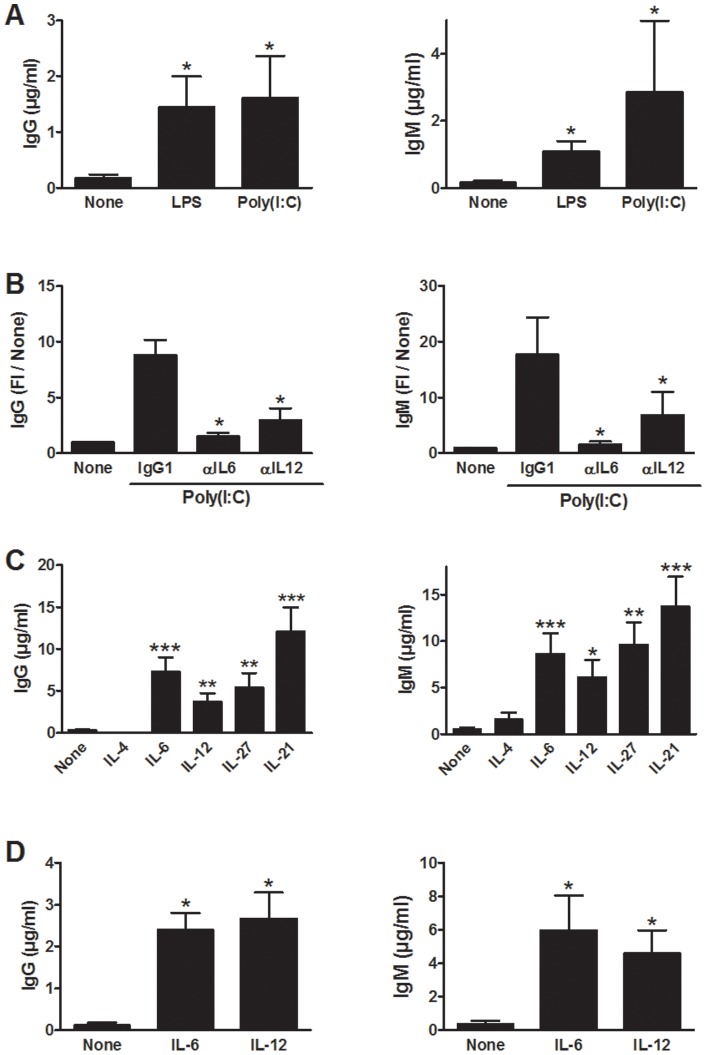
IL-6, IL-12 and IL-27 promote the differentiation of CD4 T cells helping B cells. A) Naive cord blood CD4 T cells were primed with plate bound anti-CD3 (5 µg/ml) and soluble anti-CD28 (1 µg/ml) mAbs during 72 hours in the presence of supernatant from immature moDCs activated with LPS, Poly(I∶C) or incubated with medium alone. T cells were then thoroughly washed before T/B co-culture to avoid potential carry-over effect of the DC supernatants and were incubated with anti-CD3 mAb and heterologous B cells before measuring Ig production. B) Experiments were performed as in (A) except that anti-IL-6, anti-IL-12 or control mAbs (10 µg/ml) were added to DC culture supernatants before CD4 T cell priming. C) Experiments were performed as in (A) except that recombinant cytokines were used instead of DC culture supernatants. D) Experiments were performed as in (C) except that TSST was used in the T/B co-culture instead of anti-CD3 mAb. Data are mean ± SEM of triplicates from one representation of 3 (A), of 4 (B) or 6 (C and D) independent experiments on different donors. FI/None: fold increase as compared to no cytokine. *p<0.05, **p<0.01 and ***p<0.001 as determined by paired Wilcoxon signed-rank test (A, C and D) or paired Student's t-test (B).

We first examined the capacity of soluble factors produced by activated monocyte derived dendritic cells (moDCs) to induce B cell activating CD4 T cells. In these experiments, naive cord blood CD4 T cells were stimulated polyclonally with anti-CD3 and anti-CD28 mAbs during 3 days in the presence of supernatants from LPS- or polyI:C-treated or unstimulated moDCs. Cells were then washed, irradiated and incubated with anti-CD3 mAb and heterologous B cells. Supernatants from moDCs activated by either LPS or PolyI:C strongly enhanced the capacity of naive CD4 T cells to stimulate the production of IgG and IgM by B cells (Fig. 1A), indicating that soluble factors secreted by activated DCs were sufficient to promote the differentiation of B cell activating CD4 T cells. The neutralization of IL-12 and of IL-6 during the priming of CD4 T cells reduced their capacity to stimulate IgG and IgM production (Fig. 1B).

We then evaluated the role of individual cytokines in the acquisition of B cell help capacity by naive cord blood CD4 T cells. As shown in Figure 1C, we confirmed that rIL-12 induced the differentiation of CD4 T cells stimulating IgG and IgM production [17]. CD4 T cells differentiated in the presence of rIL-6, rIL-21 or rIL-27 (STAT3-activating cytokines) also markedly promoted the production of Igs by B cells whereas no B cell stimulating activity was induced by rIL-4 (Fig. 1C). Similar results were obtained when CD4 T cells were stimulated by the superantigen TSST (toxic shock syndrome toxin) instead of anti-CD3 mAb during the co-culture with B cells (Fig. 1D). Together, these results indicate that B cell help function can be acquired by naive cord blood CD4 T cells through IL-12-dependent but also IL-12-independent pathways.

### STAT3 is critical for the acquisition of the B cell help activity induced by IL-6

**Figure 2:**
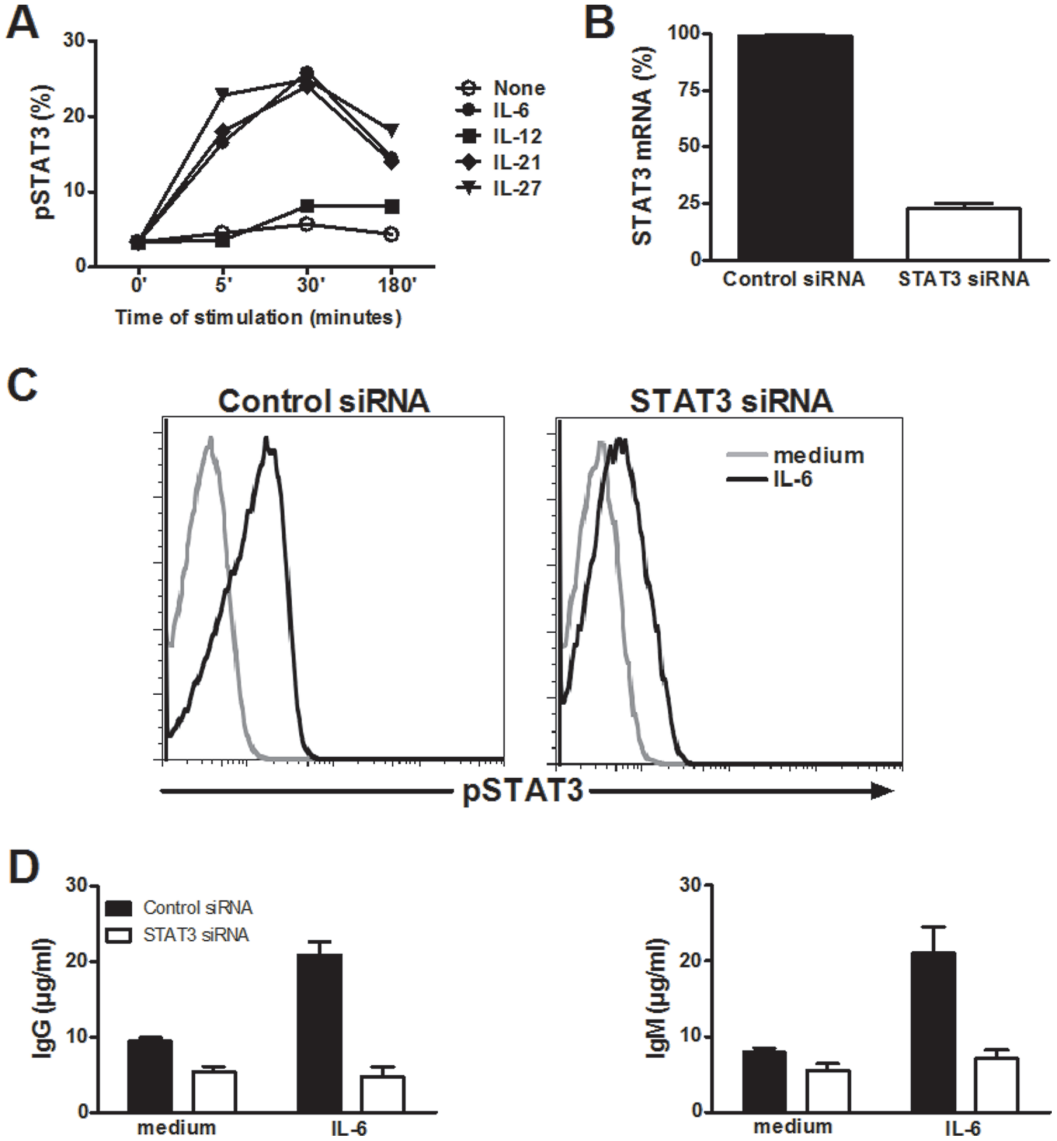
STAT3 is critical for the differentiation of CD4 T cells helping B cells induced by IL-6. A) Naive cord blood CD4 T cells were stimulated with the indicated cytokines before measuring phospho (p)STAT3 expression by flow cytometry. B and C) Naive cord blood CD4 T cells were incubated with STAT3 specific or control siRNAs in the presence of IL-2 for 48 hours. Transfected cells were stimulated for an additional 48 hours with plate-bound anti-CD3 (5 µg/ml) and soluble anti-CD28 (1 µg/ml) mAbs in the presence of rIL-6 or medium alone. We then assessed the expression of STAT3 mRNA by qRT-PCR and of pSTAT3 by flow cytometry. D) CD4 T cells were incubated in the presence of heterologous B cells before measuring the production of Ig as in Fig. 1. Data are individual results or mean ± SEM of one representative of two experiments on different donors.

As STAT3 is the major signaling molecule for IL-6, IL-21 and IL-27, we confirmed the capacity of all these cytokines to induce STAT3 phosphorylation in naive cord blood CD4 T cells. rIL-6, rIL-21 and rIL-27 induced a rapid and sustained activation of STAT3 (Fig. 2A). In contrast, rIL-12 induced a weak and late phosphorylation of STAT3. To determine the contribution of STAT3 in the acquisition of B cell help function, cord blood CD4 T cells were transfected with STAT3 siRNA before polyclonal activation in the presence of rIL-6. These conditions markedly decreased the levels of STAT3 mRNA and of pSTAT3 in CD4 T cells (Fig. 2B and 2C). Cell viability was determined before the initiation of the T/B co-cultures and was comparable following STAT3 (81 and 82% following medium or rIL-6 incubation, respectively) and control siRNA transfection (82 and 83% following medium or rIL-6 incubation, respectively). As shown in Figure 2D, STAT3 knock-down in CD4 T cells strongly reduced the capacity of rIL-6 stimulated CD4 T cells to promote the production of IgG and IgM by B cells.

### IL-21 and ICOS mediate the B cell help function of STAT3-activated CD4 T cells

**Figure 3:**
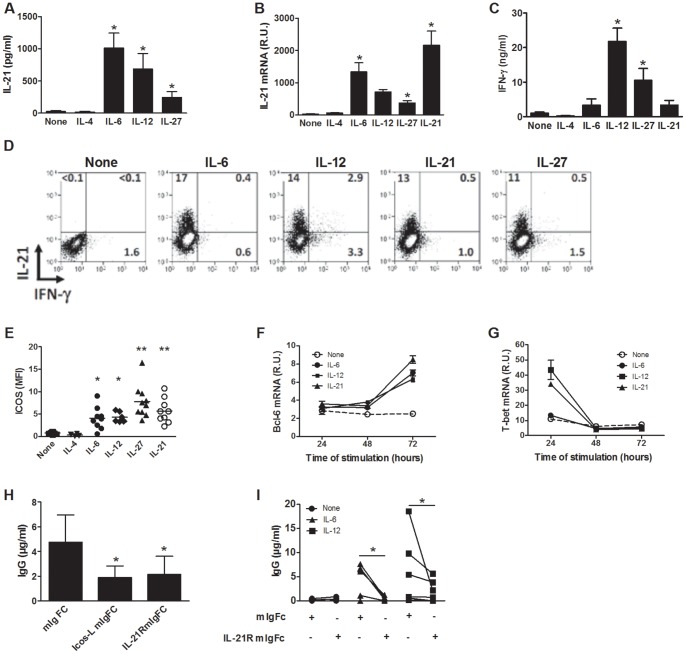
B cell help requires IL-21 production and ICOS expression. A to G) Naive cord blood CD4 T cells were stimulated with anti-CD3 and anti-CD28 mAbs in the presence of the indicated cytokines. Cytokine concentrations were determined on day 3 by ELISA (A and C), by qRT-PCR (B) and by flow cytometry (D). ICOS expression was analyzed by flow cytometry (E). Bcl-6 and T-bet expression was measured by qRT-PCR (F and G). H) Naive cord blood CD4 T cells were stimulated for 3 days with plate bound anti-CD3 (5 µg/ml) and soluble anti-CD28 (1 µg/ml) mAbs in the presence of rIL-6. Cells were then incubated with heterologous B cells and anti-CD3 mAb in the presence of ICOS-L-mIgFc, IL-21RFc or control IgFc before measuring Ig production on day 7. I) Naive cord blood CD4 T cells were stimulated for 3 days with plate-bound anti-CD3 (5 µg/ml) and soluble anti-CD28 (1 µg/ml) mAbs in the presence of rIL-6 or rIL-12 as well as IL-21RFc or control IgFc. Cells were then incubated with heterologous B cells and anti-CD3 mAb before measuring Ig production on day 7. Data are mean ± SEM of one experiment representative of two (F and G) or 6 to 10 independent experiments (A to E, H and I) except for rIL-12 on panel B (n = 4). R.U.: relative units. *p<0.05, **p<0.01 as determined by paired Wilcoxon signed-rank test or paired Student's t-test (rIL-12, panel B).

In order to determine the mechanisms involved in the B cell help function of STAT3-activated CD4 T cells, we first determined their capacity to produce IL-21 and express ICOS. Following rIL-6, rIL-12 or rIL-27 stimulation, CD4 T cells produced high levels of IL-21, both at mRNA and protein levels (Fig. 3A and 3B). Similarly, rIL-21 induced its own expression as assessed by qRT-PCR (Fig. 3B). As expected, rIL-12 and rIL-27 induced the production of IFN-γ (Fig. 3C). Intracellular staining of IL-21 and IFN-γ and flow cytometry analysis confirmed the ELISA and qRT-PCR results (Fig. 3D). Cell activation in the presence of rIL-6, rIL-12, rIL-21 or rIL-27 also increased the membrane expression of ICOS (Fig. 3E). In addition, after 72 hours of stimulation, rIL-6, rIL-12 and rIL-21 increased the expression of Bcl-6 mRNA, a key regulator of T_FH_ cell differentiation [29], [30], [31], [32] (Fig. 3F). The expression of Bcl-6 protein, determined by flow cytometry, was increased following activation with anti-CD3 and anti-CD28 but was moderately and not consistently upregulated by rIL-6, in agreement with previously published results (data not shown) [16]. T-bet mRNA was upregulated after 24 hours of stimulation of naive CD4 T cells in the presence of rIL-12 or rIL-21 but not in the presence of rIL-6 (Fig. 3G). STAT-3 activating cytokines did not upregulate CXCR5 expression by CD4 T cells, in agreement with previously published results (data not shown) [16].

As B cell help correlated with the capacity of rIL-6-stimulated CD4 T cells to produce IL-21 and to express high levels of ICOS, we further investigated the role of IL-21 and ICOS by adding soluble IL-21 receptor-Fc chimeric protein or ICOS-L-mIgFc protein during the CD4 T-B cells co-cultures. As shown in Figure 3H, blocking IL-21 or ICOS/ICOS-L interaction potently inhibited the production of IgG. In order to examine the role of IL-21 in the induction of B cell help function by rIL-6 and rIL-12, IL-21 receptor-Fc chimeric protein was added during the initial phase of CD4 T cell differentiation. As shown in Figure 3I, blocking IL-21 markedly reduced the B cell help capacity of CD4 T cells differentiated in the presence of both IL-6 and IL-12. These results suggest that IL-21 is a central autocrine factor in the differentiation of CD4 T cells helping B cells.

### Induction of IL-21 and ICOS by IL-6 is STAT3-dependent

**Figure 4:**
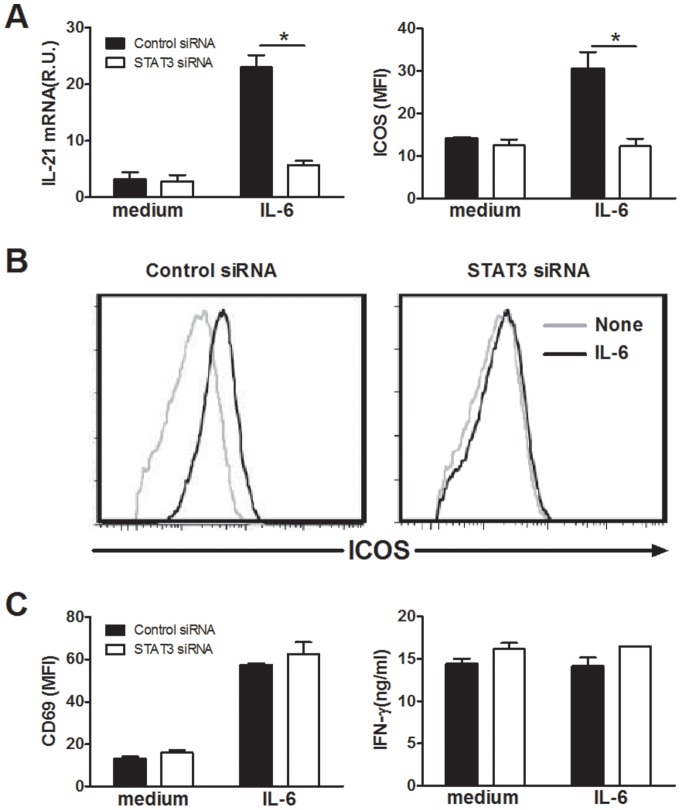
Induction of IL-21 and ICOS expression by IL-6 is STAT3-dependent. Naive cord blood CD4 T cells were transfected with STAT3 specific or control siRNAs in the presence of IL-2. After 48 h, cells were stimulated for 72 hours with plate bound anti-CD3 (5 µg/ml) and soluble anti-CD28 (1 µg/ml) mAbs in the presence of rIL-6 or medium alone before measuring IL-21 production by qRT-PCR (A), membrane expression of ICOS by flow cytometry (A and B), membrane expression of CD69 by flow cytometry and IFN-γ production by ELISA (D). Data are individual results or mean ± SEM of 3 independent experiments on different donors. R.U.: relative units. *p<0.05 as determined by Student's t-test.

To determine whether STAT3 contributed to the induction of IL-21 and ICOS expression, naive CD4 T cells were transfected with STAT3 siRNA and stimulated in the presence of rIL-6. Expression of IL-21 and ICOS was then assessed on day 3. STAT3 knock-down strongly reduced the expression of IL-21 mRNA by CD4 T cells (Fig. 4A). Only low levels of IL-21 protein were detected by flow cytometry when CD4 T cells were activated in the presence of control or STAT3 siRNA, preventing the analysis of the impact of STAT3 siRNA (data not shown). As shown in Figures 4A and 4B, STAT3 knock-down markedly reduced ICOS induction by rIL-6. As previously reported [33], rIL-12 upregulated ICOS expression by CD4 T cells and we observed that this process also involved STAT3 signalling (Fig. S1). As shown in Figure 4C, STAT3 knock-down did not affect the expression of CD69 nor the production of IFN-γ, indicating that CD4 T cells were still able to be activated and to produce cytokines. Together, these results indicate that IL-6 promotes the expression of IL-21 and ICOS by CD4 T cells through a STAT3-dependent mechanism.

### Direct recruitment of STAT3 to the proximal ICOS promoter region

**Figure 5:**
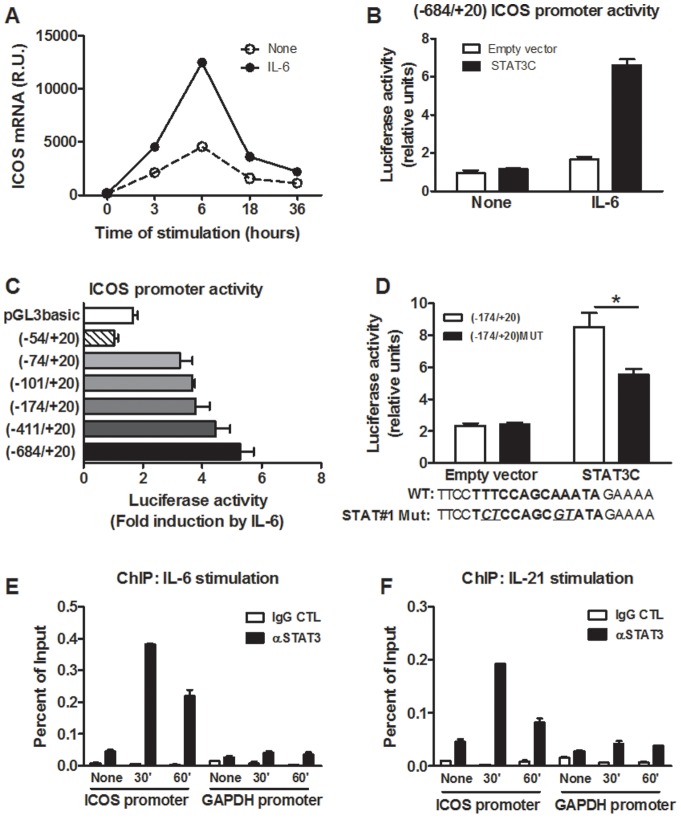
STAT3 promotes *ICOS* transcription through direct interaction with the STAT#1 binding site. A) Naive cord blood CD4 T cells were stimulated with anti-CD3 and anti-CD28 mAbs in the presence or absence of rIL-6 before measuring ICOS mRNA expression by qRT-PCR. Data are one representative out of 3 experiments on different donors. B) EL4 cells were co-transfected with the (−684/+20) ICOS reporter construct containing a luciferase element and STAT3C or control plasmids. Data are mean ± SEM of triplicates of one experiment out of 5 independent experiments. C) EL4 cells were co-transfected with the indicated reporter plasmid and STAT3C. Twenty-four hours after transfection, cells were incubated with rIL-6 or medium alone for an additional 24 hours before measuring luciferase activity. Data were normalized against unstimulated conditions for each construct and are mean ± SEM of triplicates of 4 independent experiments. D) EL4 cells were co-transfected with the (−174/+20) WT or mutated ICOS reporter construct and STAT3C. Cells were then incubated with rIL-6 or medium alone for an additional 24 hours before measuring luciferase activity. Data are mean ± SEM of triplicates of 4 independent experiments. The sequences of the STAT#1 binding site (nt −57/−43) and the mutation introduced in (−174/+20) MUT constructs are depicted. E and F) ChIP experiments. Naive cord blood CD4 T cells were stimulated with anti-CD3 mAb in the presence of rIL-6 or rIL-21. Chromatin samples were immunoprecipitated with anti-STAT3 or control antibodies. Purified DNA samples were subjected to qPCR amplification using primers encompassing the STAT#1 site from the ICOS promoter or specific for the proximal GAPDH promoter region. Data are mean ± SEM of triplicates of one representative out of two experiments on different donors. R.U.: relative units.

Despite extensive studies pointing to a crucial role for ICOS in humoral immunity, little is known about the regulation of ICOS gene expression. As the results presented above strongly suggested a role of STAT3 activating cytokines in this regulation, we examined the influence of STAT3 on ICOS gene expression. In a first set of experiments, we stimulated naive cord blood CD4 T cells in the presence or absence of rIL-6 during 3 to 36 hours and we quantified ICOS mRNA expression by qRT-PCR. IL-6 induced a strong and rapid upregulation of ICOS mRNA, with peak mRNA levels being detected at 6 hours (Fig. 5A).

In order to determine how STAT3 might directly regulate *ICOS* gene expression, we analyzed its promoter region. We cloned a 705 bp region (nt −684/+20) of the human proximal promoter as we observed that this nucleotide sequence was highly conserved in several species (Fig. S2). EL4 mouse thymoma cells were transiently transfected with a luciferase reporter construct containing this sequence and a plasmid coding for an active form of STAT3 (STAT3C). As shown in Figure 5B, STAT3C overexpression strongly increased *ICOS* promoter activity in the presence of rIL-6 whereas no effect was observed in the absence of rIL-6. This observation is consistent with the fact that STAT3C mutant is not constitutively active and requires cytokine-mediated phosphorylation on tyrosine residues [34]. Next, we generated a series of luciferase constructs containing 5′ deletions within the *ICOS* promoter region. These experiments revealed that deletion from nt −74 to −54 completely abolished the response to IL-6/STAT3C, suggesting that critical *cis*-acting elements are located within this region (Fig. 5C). We identified a putative STAT-binding site between nt −57/−43 (termed STAT#1). Mutations of the TTN5AA motif were introduced in the context of the –174/+20 reporter plasmid and were shown to affect the positive regulatory role of IL-6/STAT3C (Fig. 5D). Finally, to determine whether STAT3 physically interacts with the endogenous *ICOS* promoter region upon activation of CD4 T cells by rIL-6 and rIL-21, we performed Chromatin ImmunoPrecipitation (ChIP) experiments with primary human CD4 T cells from adult origin. Primers encompassing the STAT#1 site were used. As shown in Figures 5E and 5F, STAT3 binding to the ICOS promoter region was readily detectable in response to rIL-6 and rIL-21. Taken together, these results establish that IL-6 and IL-21-induced *ICOS* gene expression involves the direct recruitment of STAT3 to the STAT#1 site identified in the proximal promoter region.

## Discussion

The production of high affinity, isotype switched antibodies in response to vaccines or pathogens depends on B cell activation by antigen-specific CD4 T lymphocytes. Recent studies in mice have increased our knowledge on the molecular pathways involved in the acquisition of a B cell help function by CD4 T cells. In addition to the central role of Bcl-6 expression [30], [31], [32], STAT3 signaling was shown to play a crucial role in the process [12], [13], [14], [15]. In contrast, recent studies suggested that the differentiation of human CD4 T cells helping B cells primarily relies on STAT4 signaling. These reports indicated that STAT3 activating cytokines either fail or have a limited capacity to induce the production of IL-21 by naive CD4 T cells [16], [17]. Our present study unambiguously demonstrates that the STAT3 activating cytokines IL-6, IL-21 and IL-27 stimulate the capacity of CD4 T cells to help B lymphocytes and provides evidence that STAT3 is directly involved in this process, in part through the transcriptional activation of ICOS. The discordance with previously published reports could result from differences in the experimental systems used. In contrast to our study, previous reports mostly used adult peripheral blood as a source of naive T cells. Interestingly, Ma et al. reported a potent induction of IL-21 mRNA and IL-21 producing cells when cord blood naive CD4 T cells were stimulated in the presence of IL-6 or IL-21, suggesting that the source of naive CD4 T cells could influence the capacity of IL-6 to promote IL-21 production [16]. Consistent with this hypothesis, we observed that, in contrast to IL-12, IL-6 failed to promote the production of IL-21 and the capacity to help B cells when naive adult CD4 T cells were studied in our experimental settings (Fig. S3). These results suggest that STAT3 activating cytokines induce different responses in CD4 T cells recently emigrated from the thymus and in adult naive cells. Further studies are needed to test this hypothesis and to define its molecular basis.

The role of STAT3 in antibody responses *in vivo* is further illustrated by the study of patients with STAT3 mutations causing autosomal dominant hyper-IgE syndrome (AD-HIES). AD-HIES patients exhibit poor antigen-specific T cell-dependent IgG responses, reduced specific IgA levels [35], [36], [37], reduced Ag-specific effector and memory B cells and increased susceptibility to encapsulated organisms, such as S. *pneumonia* or H. *influenzae* [38], [39]. Initially, the defective antibody response was attributed to the role of STAT3 signaling in the generation of Ig secreting cells from IL-21-stimulated naive B cells [38]. Recently Ma *et al*. reported that naive CD4 T cells from AD-HIES patients are unable to acquire B cell help activity when stimulated in the presence of STAT3-activating cytokines [18]. Interestingly, the differentiation of naive CD4 T cells helping B cells induced by IL-12 was also compromised in these patients, indicating that part of the effect of IL-12 is dependent on the expression of STAT3. This possibility is supported by the results we obtained in the naive cord blood cell model and indicating that the differentiation of CD4 T cells helping B cells and the upregulation of ICOS expression induced by IL-12 involved IL-21 and STAT3, respectively.

The crucial role of ICOS in the development of GCs and in the help to B cells is well established. ICOS and ICOSL-deficient mice develop fewer and smaller GCs after immunization and have impaired T cell–dependent B cell responses. Similar impairments in B cell responses have been reported in ICOS-deficient patients [40], [41], [42]. Despite this crucial role of ICOS in T cell-dependent antibody responses, little is known about its transcriptional regulation. In mouse CD4 T cells, ICOS expression is upregulated upon TCR and CD28 engagement via the induction of NFATc2 [43]. Recently, Zhang et al reported that STAT3 is a transcriptional activator of the ICOS gene in ALK+ TCL cell lines [44]. Our study shows that upon differentiation of primary CD4 T cells in the presence of IL-6 or IL-21, STAT3 is rapidly recruited to a binding site within the proximal promoter region of the human *ICOS* locus and thereby enhances the transcriptional activity of the gene.

In addition to ICOS, IL-6 and IL-21 increased the expression of Bcl-6 mRNA by naive CD4 T cells whereas the upregulation of Bcl6 protein was relatively low and less consistent, confirming previous studies [16]. Although the role of Bcl-6 in the differentiation of human T_FH_ cells remains to be established, the increased expression of Bcl-6 mRNA further supports the notion that STAT3 activating cytokines promote the differentiation of naive human CD4 T cells derived from cord blood in T_FH_ lymphocytes. Mouse studies indicate that the differentiation and maintenance of T_FH_ cells is a complex and sequential process involving multiple signals and cellular interactions [28]. Our results therefore suggest that STAT3 signaling induces the first steps of T_FH_ cell differentiation and that additional signals and cellular interactions are probably required to establish complete cell differentiation. The capacity of STAT3 activating cytokines to initiate T_FH_ cell differentiation suggests that immunization of young infants with vaccines inducing the production of IL-6 by APCs could promote the induction of neutralizing antibodies with minimal induction of inflammatory Th1 cells. Furthermore, because ICOS plays an important role in the pathogenesis of many immune disorders, understanding the molecular mechanisms by which ICOS is regulated may create new opportunities for therapeutic interventions.

## Supporting Information

Figure S1 **Induction of ICOS expression by IL-12 is STAT3 dependent.** Naive cord blood CD4 T cells were transfected with STAT3 specific or control siRNAs. After 48 hours, cells were stimulated for an additional 72 hours with plate bound anti-CD3 (5 µg/ml) and soluble anti-CD28 (1 µg/ml) mAbs in the presence of rIL-12 before measuring membrane expression of ICOS by flow cytometry. Data are mean ± SEM of 2 independent experiments on different donors.
(TIF)

Figure S2 **Graphical representation of the proximal region of the ICOS gene.** (human hg18 chr2:204508700-204510000 corresponding to nt −1048/+252). In order to identify evolutionary conserved sequences (displayed in dark grey), the human sequence was aligned with the corresponding regions of the cow, mouse, dog and rhesus macaque genomes using ECR browser (http://ecrbrowser.dcode.org). Repetitive elements appear in light grey. The position of the potential STAT binding site (STAT#1) is indicated.
(TIF)

Figure S3 **IL-12 is a more potent inducer of adult CD4 T cells helping B cells than IL-6.** Naive adult CD4 T cells were primed during 72 hours with plate bound anti-CD3 (5 µg/ml) and soluble anti-CD28 (1 µg/ml) mAbs in the presence of rIL-6, rIL-12 or medium alone before: restimulation for 24 hours with anti-CD3 mAbs to measure IL-21 and IFN-γ production by ELISA (A and B); incubation with anti-CD3 mAb and heterologous B cells for 7 days to measure IgG production by ELISA (C).
(TIF)
