# Supplementary figures and images for: STAT3 Signaling Induces the Differentiation of Human ICOS+ CD4 T Cells Helping B lymphocytes
Source: PLoS One. 2013 Jul 26;8(7):e71029. doi: 10.1371/journal.pone.0071029 (PMC3724802; doi:10.1371/journal.pone.0071029)

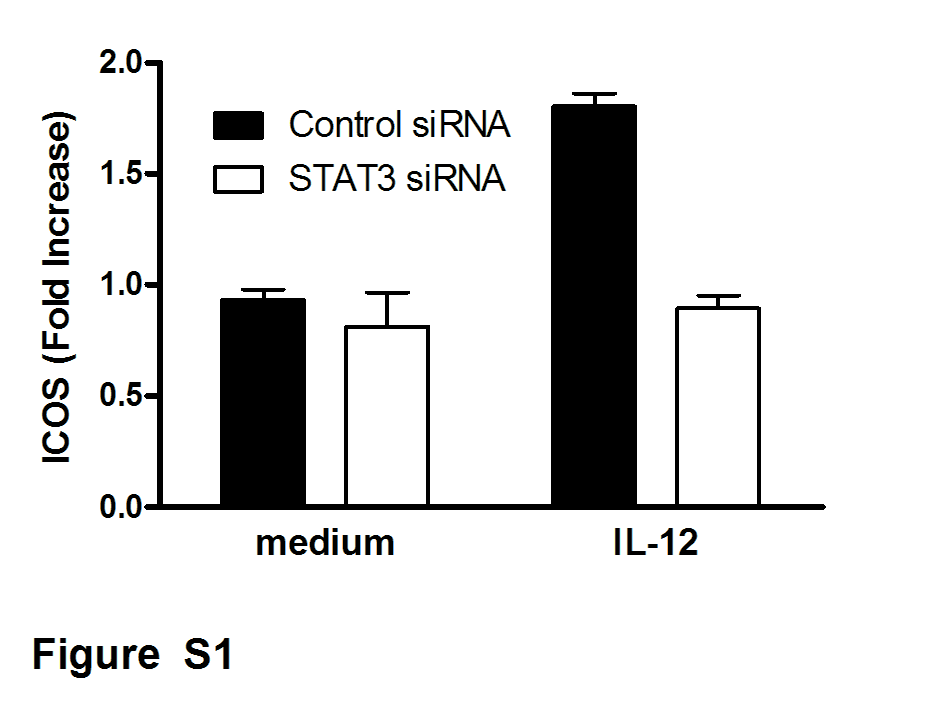

Supplement: Figure S1 — Induction of ICOS expression by IL-12 is STAT3 dependent. Naive cord blood CD4 T cells were transfected with STAT3 specific or control siRNAs. After 48 hours, cells were stimulated for an additional 72 hours with plate bound anti-CD3 (5 µg/ml) and soluble anti-CD28 (1 µg/ml) mAbs in the presence of rIL-12 before measuring membrane expression of ICOS by flow cytometry. Data are mean ± SEM of 2 independent experiments on different donors. (TIF) [file pone.0071029.s001.tif]

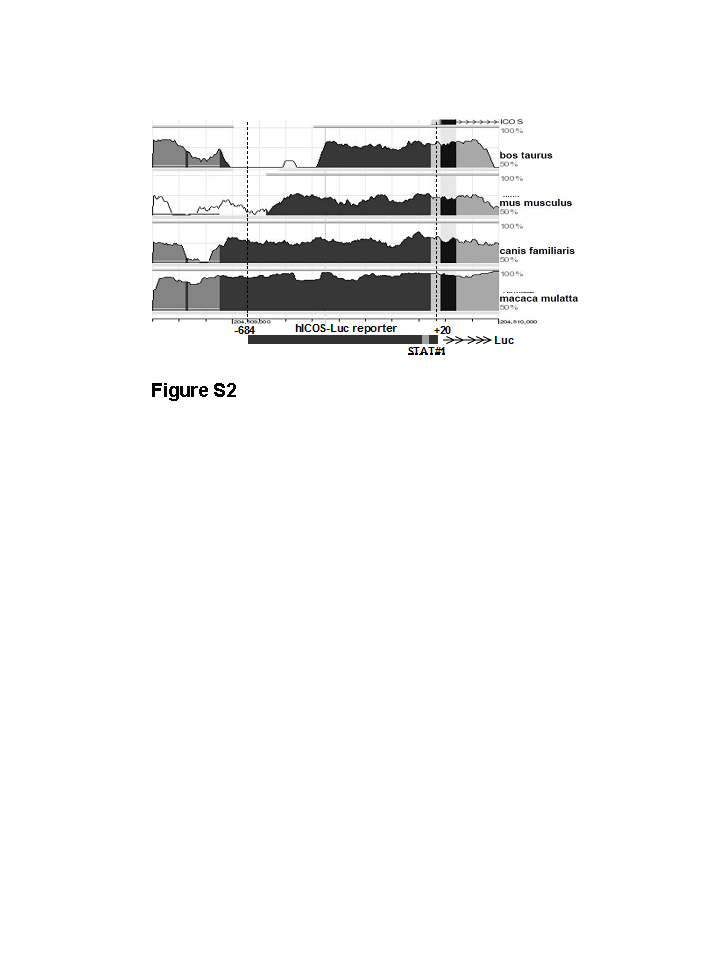

Supplement: Figure S2 — Graphical representation of the proximal region of the ICOS gene. (human hg18 chr2:204508700-204510000 corresponding to nt −1048/+252). In order to identify evolutionary conserved sequences (displayed in dark grey), the human sequence was aligned with the corresponding regions of the cow, mouse, dog and rhesus macaque genomes using ECR browser (http://ecrbrowser.dcode.org). Repetitive elements appear in light grey. The position of the potential STAT binding site (STAT#1) is indicated. (TIF) [file pone.0071029.s002.tif]

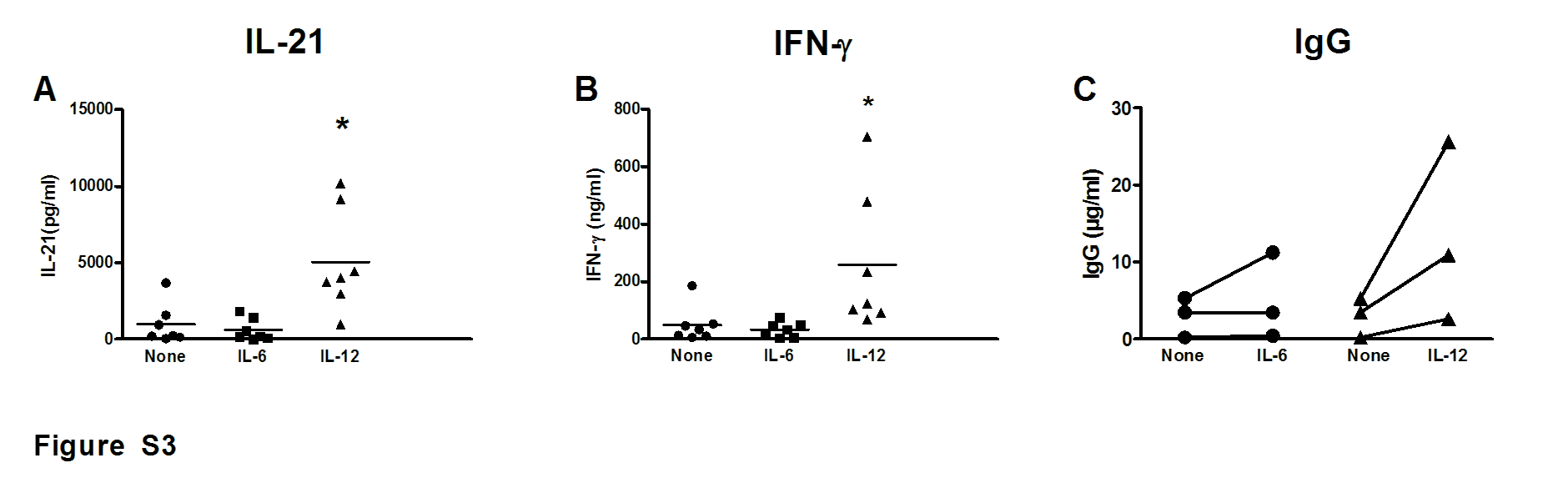

Supplement: Figure S3 — IL-12 is a more potent inducer of adult CD4 T cells helping B cells than IL-6. Naive adult CD4 T cells were primed during 72 hours with plate bound anti-CD3 (5 µg/ml) and soluble anti-CD28 (1 µg/ml) mAbs in the presence of rIL-6, rIL-12 or medium alone before: restimulation for 24 hours with anti-CD3 mAbs to measure IL-21 and IFN-γ production by ELISA (A and B); incubation with anti-CD3 mAb and heterologous B cells for 7 days to measure IgG production by ELISA (C). (TIF) [file pone.0071029.s003.tif]
